# Acute myeloid leukemia: current progress and future directions

**DOI:** 10.1038/s41408-021-00425-3

**Published:** 2021-02-22

**Authors:** Hagop Kantarjian, Tapan Kadia, Courtney DiNardo, Naval Daver, Gautam Borthakur, Elias Jabbour, Guillermo Garcia-Manero, Marina Konopleva, Farhad Ravandi

**Affiliations:** Department of Leukemia, MD Anderson Cancer Center, Houston, TX USA

**Keywords:** Prognosis, Health services

## Abstract

Progress in the understanding of the biology and therapy of acute myeloid leukemia (AML) is occurring rapidly. Since 2017, nine agents have been approved for various indications in AML. These included several targeted therapies like venetoclax, FLT3 inhibitors, IDH inhibitors, and others. The management of AML is complicated, highlighting the need for expertise in order to deliver optimal therapy and achieve optimal outcomes. The multiple subentities in AML require very different therapies. In this review, we summarize the important pathophysiologies driving AML, review current therapies in standard practice, and address present and future research directions.

## Introduction

**Fig. 1:**
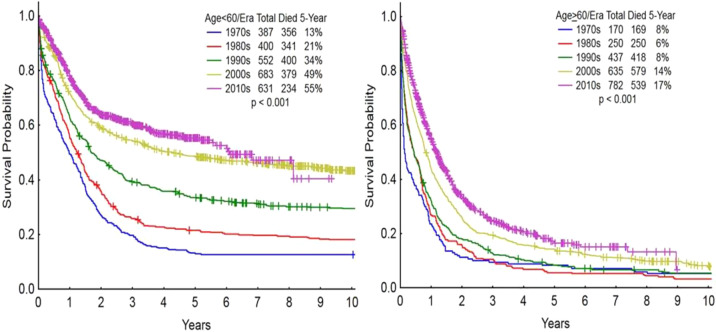
Survival of de novo acute myeloid leukemia at MD Anderson (1970–2017) by Age and Treatment Era: Left panel: age<60 years; Right panel: age 60+ years.

Progress in understanding the pathophysiology and improving the therapy of acute myeloid leukemia (AML) is now occurring at a rapid pace. The discovery of the activity of cytarabine (ara-C) and of anthracyclines in AML, and combining them in the 1970’s, into what is known as the “3 + 7 regimen” (3 days of daunorubicin + 7 days of cytarabine), has long been considered the standard of care, resulting in long-term cures of 30 to 40% among younger patients with AML^1–5^. The earlier studies focused on patients usually up to the age of 50–55 years, and reported 5-year survival rates of 40–45%. Later studies including patients up to the age of 60 years reported 5-year survival rates of 30–35%. These intensive chemotherapy regimens, applied commonly in older patients (age 60 years and older), resulted in 5-year survival rates of <10–15%^6,7^. Figure 1 shows the MD Anderson outcomes in AML in younger and older patients from 1970 to 2018.

Unraveling the heterogeneity of AML at the clinical, cytogenetic, and molecular levels allowed improved prognostic and predictive abilities and led to the development of selected therapies for AML subsets. Chemotherapy-free regimens consisting of all trans-retinoic acid (ATRA) and arsenic trioxide in acute promyelocytic leukemia (APL) resulted in cure rates of 90%^8–12^. In core-binding factor (CBF) AML, adding gemtuzumab ozogamicin (CD33-targeted monoclonal antibody conjugated to the calicheamicin payload) to high-dose cytarabine-based chemotherapy increased the long-term survival rate from 50% to 75+%^13–17^.

Research efforts in the last decade have expanded the pathophysiologic-molecular subsets of AML, through identification of prognostic, predictive, and targetable molecular abnormalities^18–25^. Ongoing studies and recently approved agents in AML of particular interest include: (1) Combinations of epigenetic therapy with hypomethylating agents (HMAs; azacitidine, decitabine) and venetoclax in older patients (or patients unfit for intensive chemotherapy); and combinations of intensive chemotherapy and venetoclax in younger/fit patients. (2) The addition of fms-like tyrosine kinase 3 (FLT3) inhibitors (gilteritinib, midostaurin, sorafenib, quizartinib, crenolanib, others) to intensive chemotherapy, or to HMA/low-intensity therapy in *FLT3*-mutated AML. (3) The addition of IDH inhibitors (IDH1 inhibitor ivosidenib; IDH2 inhibitor enasidenib) and/or venetoclax in *IDH1/2-* mutated AML. (4) Investigations of the roles of APR246 (TP53 modulator) and of magrolimab (anti-CD47 monoclonal antibody enhancing the macrophage-mediated phagocytosis) in *TP53*-mutated AML. (5) Exploring the role of menin inhibitors in mixed-lineage leukemia (*MLL1*)-rearranged acute leukemia. (6) Investigations of combined small-molecule targeted therapies, with or without standard intensive chemotherapy or HMAs (+/− venetoclax; at the expense of worsening myelosuppression), in order not only to prolong survival, but also to improve the potential cure rates in previously incurable AML subsets. (7) Establishing maintenance therapy as an important strategy in AML (as it is in acute lymphoblastic leukemia [ALL]). (8) Developing oral anti-AML therapy (e.g., oral decitabine, oral azacitidine) to replace and improve upon the effects of parenteral therapies. (9) Approaches to enhance T-cell immune responses to AML (as done in ALL) with T-cell engagers (BiTEs), checkpoint inhibitors, and chimeric antigen receptor (CAR)-T-cell approaches.

Many AML experts ascribe to the 3 + 7 regimen as the AML standard of care today; others may not. We will discuss the results with 3 + 7 and put them into context with the more recent combined modality regimens, which may be superior. The nihilistic mood that prevailed in the AML community until 2015 has lifted, particularly with research resulting in the FDA approvals of multiple agents for AML since 2017 (Table 1). It is interesting to compare this AML review to the one published in 2016^1^, to appreciate the previous “bare cupboard” in AML research and the tremendous progress over such a short period of time. Prior to 2017, some decisions may have temporarily slowed progress in AML. One example is the voluntary withdrawal of gemtuzumab ozogamicin (GO) by the manufacturer (June 2010) from clinical use in the United States based on a negative trial by the Southwest Oncology Group (SWOG)^16^. This was remedied with the GO re-approval in 2017 at a lower dose to minimize toxicity, based on a meta-analysis of five randomized frontline trials in AML clearly demonstrating benefit^17^. The use of GO is now particularly important in the therapy of CBF AML and APL. The second example was the non-approval of decitabine in the US for frontline therapy of older patients with AML (approved in Europe)^26,27^. Low-intensity HMA therapy with decitabine and azacitidine-based regimens is now the most common form of treatment among older (or unfit for intensive chemotherapy) patients with AML^26,28^. A third possible example is the non-submission of vosaroxin for FDA approval for the therapy of AML first salvage^29^. Vosaroxin may have offered a non-cardiotoxic form of topoisomerase-II inhibitor therapy.

**Table 1:** Recent Food and Drug Administration Drug Approvals (since 2017) in acute myeloid leukemia.

Treatment (approval date)	Description	Indication
Midostaurin (April 2017)	Multikinase FLT3 inhibitor	Newly diagnosed *FLT3*-mutated (as detected by FDA-approved test) AML, in combination with standard cytarabine and daunorubicin induction and cytarabine consolidation
Gemtuzumab ozogamycin (September 2017)	Anti-CD33 antibody–drug conjugate	Adults with newly diagnosed CD33-positive AML; refractory-relapsed CD33-positive AML in patients ≥ 2 years of age
CPX-351 (August 2017)	Liposomal cytarabine and daunorubicin at a fixed 5:1 molar ratio	Newly diagnosed therapy-related AML, secondary AML or AML with myelodysplasia-related changes
Glasdegib (November 2018)	Hedgehog pathway inhibitor	Newly diagnosed AML aged ≥ 75 years or with co-morbidities that preclude the use of intensive induction chemotherapy (in combination with low-dose cytarabine)
Venetoclax (November 2018)	BCL-2 inhibitor	In combination with azacitidine or decitabine, or low-dose cytarabine in newly diagnosed AML aged ≥ 75 years or with co-morbidities that preclude the use of intensive induction chemotherapy
Enasidenib (August 2017)	IDH2 inhibitor	Relapsed or refractory *IDH2-* mutated AML (as detected by FDA-approved test)
Ivosidenib (July 2018) (May 2019)	IDH1 inhibitor	1. Relapsed or refractory *IDH1*-mutated (susceptible mutation, as detected by FDA-approved test) AML. 2. First line treatment of *IDH1*-mutated AML (as detected by FDA-approved test), patients ≥ 75 years old or ineligible to receive intensive chemotherapy.
Gilteritinib (November 2018)	FLT3 inhibitor	Patients with relapsed or refractory *FLT3*-mutated AML (as detected by FDA-approved test)
CC-486 (September 2020)	Oral azacitidine hypomethylating agent (30% absorption) approved at 300 mg daily × 14 every month	Continued treatment of adult patients with AML who achieved first complete remission or complete remission with incomplete blood count recovery following intensive induction chemotherapy and who are not able to complete intensive curative therapy
Oral Decitabine-cedazuridine (July 2020)	Oral hypomethylating agent (100% absorption)	Alternative to parenteral HMAs decitabine for the treatment of adults with MDS (pretreated/untreated; de novo/secondary) or CMML

In this review, we discuss progress in AML research, outline the MD Anderson approaches in 2020, and explore investigational strategies over the coming years.

## Cytogenetic and molecular abnormalities

Acute myeloid leukemia has diverged from being considered as one acute leukemia entity to become a heterogeneous constellation of AML subentities characterized by diverse pathophysiologic, clinical, cytogenetic, and molecular profiles that benefit from individualized selective therapies and have vastly different outcomes.

The cytogenetic-molecular entities in AML are outlined in Table 2^30–50^. These include APL with its characteristic translocation 15;17 [t(15;17) (q22,q21)]; inversion 16 [inv 16(p13; q22)] or t(16;16) (p13;q22) and t(8;21)(q22;q22,), together referred to as CBF AML; diploid karyotype (about 40–50% of patients); complex karyotype (three or more chromosomal abnormalities); others.

**Table 2:** Cytogenetic-molecular entities in acute myeloid leukemia (NCCN classification).

NCCN	Cytogenetics	Molecular abnormalities
Better risk	-Inversion (16) or translocation (16;16) -Translocation (8;21) -Translocation (15;17)	Normal cytogenetics: *NPM1* mutation in the absence of *FLT3*-ITD or *FLT3*-ITD low allelic ratio; isolated biallelic *CEBPA* mutation
Intermediate risk	-Normal cytogenetics -Trisomy 8 alone -Translocation (9;11) -Other non-defined	- Translocation (8;21), inversion (16), translocation (16;16): with *c-KIT* mutation - *NPM1*-mutated and FLT3-ITD mutated (high allelic ratio) - NPM1-wild type and FLT3*-* wild type - NPM1-wild type and *FLT3-*ITD mutated (low allelic ratio)
Poor risk	-Complex (≥3 clonal chromosomal abnormalities) -Monosomal karyotype: -5, 5q-, 7, 7q- -11q23—non translocation (9:11) -Inversion (3), translocations of (3;3) -Translocation (6;9) or (9;22)	- *TP53* mutation - *RUNX1* mutation - *ASXL1* mutation - NPM1-wildtype and *FLT3-*ITD mutated (high allelic ratio)

Notes related to the NCCN Risk classification:

(1) The NCCN classification is applicable to younger patients with AML (age up to 60–65 years old) and in de novo AML. Older patients with AML and patients with secondary (progression to AML from myelodysplastic syndrome, particularly if treated; or from myeloproliferative neoplasm) or therapy-related AML have significantly poorer outcome within each of the NCCN risk categories (the exception being possibly APL).

(2) At MD Anderson, all translocations involving 11q23 are considered adverse. Also, in updated analyses, a translocation (9;11) may be intermediate risk only in de novo younger AML (but not in older or secondary/therapy-related AML).

(3) The differential effect of mutations is particularly notable in patients with diploid or intermediate risk karyotype, but not in patients with better or poor risk karyotypes.

Low allelic ratio is < 0.5; high allelic ratio is ≥ 0.5.

**A**dapted from National Cancer Centers Network (NCCN). Accessed October 9, 2020. https://www.nccn.org/

*NCCN* National Cancer Centers Network.

Molecular subsets also define prognosis and are therapeutically targetable. Among patients with a diploid karyotype, single mutations and mutation combinations interact differently, in sometimes intricate balancing acts. For example, a mutation of nucleophosmin-1 (*NPM1*) without a *FLT3* mutation is associated with a more favorable outcome. If a *FLT3* mutation, particularly *FLT3* internal tandem duplication *(FLT3-*ITD*)*, is present (about 50% of patients with a diploid karyotype and *NPM1* mutation), then the outcome was worse historically, and largely dependent on the *FLT3* allelic ratio (AR). In newly diagnosed *FLT3-*mutated AML, the AR of *FLT3-*ITD to FLT3*-*wild type strongly influenced outcome in several studies of chemotherapy-based therapies that did not include FLT3 inhibitors^34–36^. The *FLT3-*ITD AR is defined as the ratio of the area under the curve of “*FLT3-*ITD” divided by the area under the curve of “FLT3*-*wildtype” using a semi-quantitative DNA fragment analysis^30^. A higher *FLT3-*ITD AR (generally defined as ⩾0.5) is associated with worse survival than lower ratios, likely reflecting increased FLT3 dependency in cases with high ARs. This may change with the incorporation of FLT3 inhibitors into AML chemotherapy and into post stem cell transplantation (SCT) maintenance. Mutations, including *ASXL1, RUNX1, TP53*, and others may also associate with outcome differences. Several molecular mutations are potentially targetable (Table 3)^30–51^.

**Table 3:** Clinically relevant mutations in acute myeloid leukemia.

Mutation	% Incidence (with diploid karyotype)	Comments
*FLT3-*ITD	20 (30–35)	Adverse prognosis—high allelic ratio is an indication for allogeneic SCT; adding FLT3 inhibitors as post SCT maintenance. Outcome may change with the addition of FLT3 inhibitors to chemotherapy (sorafenib, midostaurin, gilteritinib)
*FLT3-*TKD	5–10	Prognostic significance uncertain; response to Type I FLT3 inhibitors like gilteritinib and midostaurin.
*NPM1*	30 (40–50)	*FLT3* wild-type *NPM1-*mutated = favorable prognosis. Older patient + *NPM1*-mutated AML = more sensitive to cytarabine and hypomethylating agents + venetoclax
*CEBPA*	< 5	Biallelic mutations = better prognosis (without concomitant unfavorable mutations)
*DNMT3A*	20 (30–35)	Associated with *NPM1* and *FLT3*-ITD. Adverse prognosis, especially with concomitant *FLT3* mutations; epigenetic modulation
*RUNX1*	10	Adverse prognosis
*ASXL1*	10–15	Adverse prognosis
*KIT*	5	Incidence higher in CBF-AML;? unfavorable outcome in CBF-AML (? need for c-KIT inhibitors). Possible benefit from addition of gemtuzumab ozogamicin
*NRAS*	10–15	40–50% of inversion16 AML; no definite prognostic association; may be a mechanism of resistance to BCL2, IDH, and FLT3 inhibitors (especially Type I) at the time of relapse.
*IDH2*	10–20 (20–30)	Therapy with enasidenib and/or venetoclax-based combinations
*IDH1*	7–10 (10–15)	Therapy with ivosidenib and/or venetoclax-based combinations
*TET2*	10–15	Adverse prognosis; epigenetic modulation
*TP53*	2–20	High incidence (70%) in complex karyotype; very adverse prognosis. Limited benefit of intensive chemotherapy among those with allelic frequency of ≥ 40%. Investigational approaches should be considered (i.e., APR-246; magrolimab and other anti-CD47 antibodies)

Prognostic impact of mutations mostly in the context of normal karyotype.

Next-generation sequencing identified multiple recurrent somatic mutations in >90% of patients with AML^21,52^. Frequently mutated genes (frequency >5%) are *FLT3, NPM1, DNMT3A, IDH1, IDH2, TET2, RUNX1, p53, NRAS, CEBPA, WT1*^21,24,52^. Based on functional analysis and known pathways, these are routinely grouped into biologic- functional categories: myeloid transcription-factor fusions or mutations; *NPM1* mutations; tumor-suppressor gene mutations; epigenome-modifying gene mutations; activated signaling-pathway gene mutations; cohesin-complex gene mutations; and spliceosome-complex gene mutations. These mutations exhibit shared co-occurrences or exclusive dissociations that help identify AML pathways of clonal dominance and shifts that would result into more rational targeting therapies.

Translating to clinical practice, the important molecular subsets are based on the identification of a *FLT3* mutation (30% of AML), *NPM1* mutation (40–50% of normal karyotype AML), isocitrate dehydrogenase 1 or 2 (*IDH1/2*) mutations (20% of AML), and *TP53* mutations (2 to 20% of AML).

Patients with *NPM1*-mutated AML have a more favorable prognosis; those with *FLT3-*ITD mutations have a poor prognosis, especially among patients with high *FLT3* ARs and in the absence of *NPM1* mutation. Patients with diploid karyotype AML (without adverse mutations such as *TP53*, or *ASXL1*) and biallelic *CEBPA* mutations (2% or less of AML) have a favorable prognosis^5^.

The *FLT3* mutations, including *FLT3-*ITD and *FLT3-*tyrosine kinase domain (TKD) point mutations (D835 most common), can now be targeted with FLT3 inhibitors. Midostaurin and gilteritinib are type I FLT3 inhibitors and suppress both *FLT3-*ITD and *FLT3-*TKD mutations. Sorafenib and quizartinib are type II FLT3 inhibitors that target only *FLT3-*ITD.

The *IDH1/2* mutations can be targeted with novel IDH inhibitors, ivosidenib, which targets *IDH1* mutations, and enasidenib, which targets *IDH2* mutations. The *IDH1/2* mutations also generate AML dependence on BCL-2 for survival, rendering them responsive to venetoclax-based therapy^53^.

Mutations of epigenetically related molecular events (*DNMT3A*, *IDH1/2*, *TET2, ASXL1*, and *MLL1*) may suggest the possible benefit of epigenetic-targeted therapy.

In CBF AML, mutations in *c-KIT* may be associated with worse outcome in some studies^47–50^, but not with fludarabine-cytarabine-GO-based regimens^12–14^. Investigating the addition of a potent c-KIT inhibitor (avapritinib, dasatinib) to chemotherapy in *c-KIT*-mutated CBF AML is of interest^50,51^.

Mutations and/or deletions of the tumor-suppressor gene *TP53* (located on the short arm of chromosome 17) occur in 2–20%, are more common in older patients and patients with secondary or therapy-related AML, and are associated with complex cytogenetics^38–41^. In a study of 293 patients, 53 (18%) had *TP53* mutations; these were associated with complex karyotype (*p* < 0.001) and with abnormalities of chromosomes 17 and 5 and/or 7, and with a low CR rate and short survival^39^. Most patients with *TP53* mutations may not benefit from intensive chemotherapy and may have similar or improved outcomes and less toxicity with lower intensity approaches^40,54^. The variant allelic frequency (VAF; percent mutated/total) of *TP53* mutations may help select patients who would not benefit from intensive induction therapy. Novel strategies like APR-246 or magrolimab have shown promise.

Patients with the cytogenetic-molecular subset of “mixed-lineage leukemia” (translocations involving 11q23; *MLL1*, *KMT2A* rearrangement) may respond well to the novel menin inhibitors (SNDX-5613, KO-539, others)^55^.

Translocations involving chromosome 3q26.2 (*EVI1*), location of the MECOM (MDS1 and EVI1 complex locus) gene, have an extremely poor outcome with standard chemotherapy^46^. Additional mutations associated with adverse outcomes are *DNMT3A*^42,43^, *ASXL1*, *RUNX1*^44,45^, and others^40–44^.

## Measurable residual disease in complete remission

Measuring residual disease in AML in complete remission (CR) is now part of the standard of care in AML^56–62^. The detection of measurable residual disease (MRD) at the time of morphologic CR is associated with a higher relapse rate and with worse survival in AML. Measurable residual disease has been commonly investigated using two methodologies, multi-color flow-cytometric measurements of MRD (MFC-MRD), and molecular quantification of residual disease^56–61^.

Polymerase chain reaction (PCR) measure of residual molecular disease is routinely used to monitor quantitatively unique AML-defining translocations and mutations in APL, CBF AML, *NPM1-*mutated AML, and now expanding to other molecular subsets (*IDH1/2* and *FLT3* mutations). In APL, PCR quantification of promyelocytic leukemia-retinoic receptor alpha (*PML-RAR alpha*) may detect early molecular relapse^63^. The same is true for CBF AML. Inversion 16 and t (16; 16) result in the formation of the CBF beta/myosin heavy chain 11 (*CBFB/MYH11*) fusion gene. The t (8; 21) leads to the formation of the Runt-related transcription factor 1 [*RUNX1*]/*RUNX1T1* (*RUNX1*/*RUNX1T1*) fusion gene. Detection of molecular fusion genes MRD by quantitative PCR in CBF AML (especially AML with inversion 16) predicts for relapse^64,65^. Interestingly, patients with t (8; 21) may have persistent MRD at levels below 0.1%, but still remain in durable complete remissions and possibly cured. Among patients with non-CBF non-APL AML, monitoring mutations by next-generation sequencing is informative when possible, for example in patients with *NPM1* mutations^66,67^. Combining MFC and molecular PCR studies may improve on the capability of MRD studies to predict for relapse^56^. Better outcomes are reported in *FLT3*-mutated and *IDH*-mutated AML with molecular MRD clearance.

Measurable residual disease in CR indicates worse prognosis due to a higher risk of relapse. This should lead to consideration of therapeutic interventions. In APL, therapy at the time of molecular relapse improved survival compared with therapy at the time of hematologic relapse^63^. Allogeneic SCT for persistent MRD in CR in CBF AML improved survival compared with continuation of standard therapy^65^. Important interventions in AML with MRD in CR may include allogeneic SCT; investigational approaches with more intensified chemotherapy regimens, or with HMAs (parenteral or newly approved oral formulations) plus venetoclax; targeted therapy combinations when indicated for particular molecular abnormalities (FLT3 or IDH inhibitors); antibody therapies (e.g., CD123 or CD33 monoclonal or BiTEs); or immune therapies (e.g., checkpoint inhibitors). However, the persistence of DTA mutations in CR (mutations in *DNMT3A, TET2, ASXL1*) does not predict for relapse^56^.

## Treatment of AML

The heterogeneous group of AML disorders requires different selective therapies. Next, we will discuss the treatment of the highly curable leukemias, APL and CBF AML; the different therapeutic approaches in younger and older patients with AML; and the addition of the novel targeted therapies (venetoclax, FLT3 inhibitors, and IDH inhibitors) to standard therapies.

## Acute promyelocytic leukemia

Acute promyelocytic leukemia represents 5–10% of AML and is defined by the cytogenetic abnormality t (15; 17), which results in the *PML-RAR alpha* fusion oncogene and its encoded oncoprotein. The PML-RAR α oncoprotein acts as a dominant negative inhibitor of wild-type RAR α, causing a maturation block and the clinical-pathologic picture of APL.

Combinations of anthracyclines and cytarabine first established the potential cure rate of 30–40% in APL^68,69^. The early mortality from disseminated intravascular coagulopathy (DIC) and bleeding with anthracyclines-cytarabine was significant, about 10–20%. The added anti-APL efficacy of high-dose cytarabine and maintenance chemotherapy (POMP) was modest at best^69^.

In the late 1980s and early 1990s, the major anti-APL efficacies of ATRA and arsenic trioxide were discovered. Gemtuzumab ozogamicin was also highly effective in APL^70^. The most potent anti-APL drugs are arsenic trioxide, followed by ATRA, GO, and anthracyclines.

Based on the single-agent anti-APL efficacies of ATRA and arsenic trioxide^71^, ATRA was initially added to chemotherapy during both induction and/or consolidation^72–74^, and arsenic trioxide was investigated initially in APL relapse and later as consolidation therapy^75^. Comparative studies showed that the addition of ATRA to chemotherapy during induction and/or consolidation improved survival^71–73^, and that the addition of arsenic trioxide during consolidation in CR also improved event-free survival (EFS). In the late 1990’s, the combination of idarubicin (or other anthracyclines) and ATRA (AIDA regimen) became standard of care in APL^76^.

### Chemotherapy-free regimens: ATRA and arsenic trioxide

The MD Anderson group first investigated the use of non-chemotherapy regimens including ATRA, arsenic trioxide and GO, and demonstrated the high efficacy of this strategy^8^. Randomized studies confirmed the superiority of ATRA plus arsenic trioxide over AIDA in low and intermediate risk APL^11,77^. A SWOG study also demonstrated the efficacy and safety of ATRA with arsenic trioxide and GO in high risk APL^78^. With the ATRA plus arsenic trioxide regimens, the CR rate is 90+% and the cure rates 80+%. Induction mortality from DIC is low (about 5%). Resistant disease is extremely rare, except in molecular variant-APL (translocations between chromosome 11 and 17 [*PLZF-RAR alpha*], or between chromosome 5 and 17).

**I**mportant considerations in APL management are: (1) Granulocyte-colony stimulating growth factors (filgrastim, pegfilgrastim) should *never* be used in APL, as it is the one leukemia where granulocyte growth factors may induce a drastic increase in APL progression, and trigger fatal DIC^79^. (2) Watch for fluid overload (often confused with “differentiation syndrome”). This is related to ATRA and arsenic trioxide, as well as the use of high-volume blood product transfusions (fresh frozen plasma) to prevent the complication of consumptive coagulopathy. These complications are best managed by holding ATRA-arsenic trioxide therapy briefly and with aggressive diuresis^80^. (3) The development of a “differentiation syndrome” with possible multi-organ failure; this requires the use of prophylactic steroids during induction (together with antibiotics and antifungal prophylaxis). (4) Among patients with CNS bleeding at diagnosis, the risk of CNS leukemia may increase; two intrathecal cytarabine injections in CR may eliminate this rare complication.

The MRC comparative trial investigated a lower dose schedule of arsenic trioxide 0.3 mg/kg on Days 1–5 during week 1, then 0.25 mg/kg twice weekly in weeks 2–8 of Course 1 followed by 4 consolidations courses (63 arsenic trioxide doses)^77^.

Oral formulations of arsenic trioxide are under investigation; these would render the treatment of APL more convenient, particularly during the longer term consolidation^81,82^

**Fig. 2:**
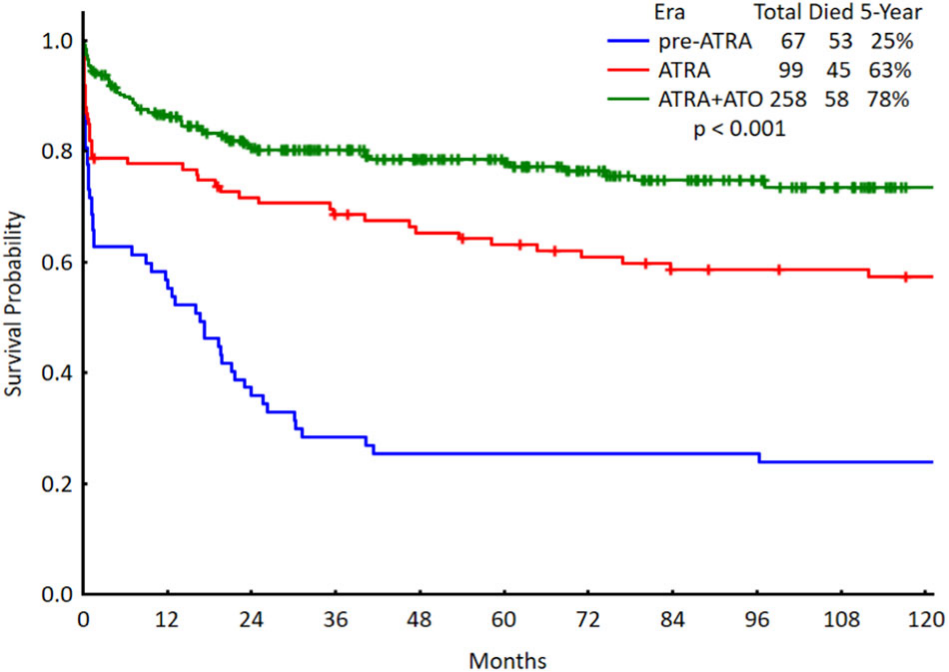
Survival of acute promyelocytic leukemia at MD Anderson (1970–2020).

Figure 2 shows the MD Anderson results in APL, and the significant outcome improvement in the era of ATRA and arsenic trioxide.

## Core-binding factor acute myeloid leukemia

The CBF AMLs include the subsets with chromosomal abnormalities involving inversion 16/t (16; 16) or t (8; 21). These constitute 10–15% of adult AML cases.

**Fig. 3:**
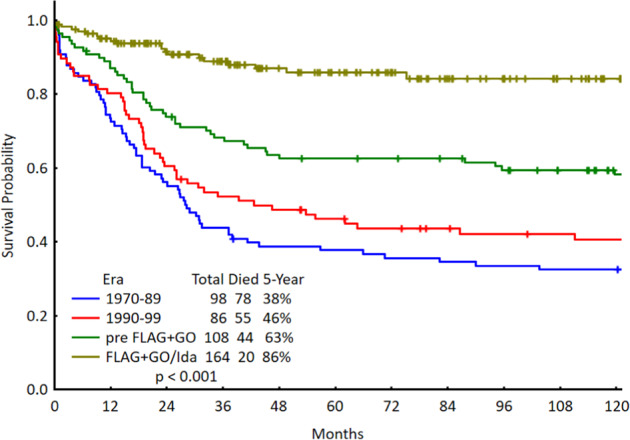
Survival of core-binding factor acute myeloid leukemia at MD Anderson (1970–2020).

The use of established chemotherapy drugs in optimized combinations has gradually improved the cure rates in CBF AML from <50% to about 75%^13–17^. Historically, CBF AML was treated with cytarabine plus anthracycline induction chemotherapy followed by 1–4 high-dose cytarabine consolidations. The cure rates were 30–40% with one consolidation versus 50+% with 3–4 consolidations^83,84^. Using induction- consolidation courses of high-dose cytarabine combinations with fludarabine and idarubicin, and the addition of GO 3 mg/m^2^ × 1 during induction and consolidation (comparative SWOG and MRC studies) resulted in estimated 5-year survival rates of 75+% in CBF AML^13–17^. At MD Anderson, we currently use fludarabine, high-dose cytarabine and GO (i.e., FLAG-GO) during induction and consolidations, for a total of up to six courses, and modify therapy with the addition of maintenance for persistent MRD at the completion of therapy. The results were better when GO replaced idarubicin. The 5-year survival rates were 80% in both inversion 16 and t (8; 21) AML (Fig. 3)^3,14^. The MRC trials using the fludarabine, high-dose cytarabine and idarubicin combination (FLAG- IDA regimen) +/− GO also reported cure rates of 80+% in CBF AML^15^. In a meta-analysis of five studies, adding GO to standard induction-consolidation therapy improved survival from 50 to 75%^17^.

Today, GO should always be added to the standard chemotherapy in CBFAML. The new regimens utilizing fludarabine, high-dose cytarabine and GO, with or without idarubicin, may be better, producing cure rates of 75–80+%^14,15^.

The CBF AML often exhibits co-occurrence of mutations in *FLT3* (15–20%), *c-KIT* (29–30%), *RAS* (30–50%), and others. Some studies report *c-KIT* or multiple mutations to be associated with worse prognosis^47–49^. This has not been our experience with the FLAG-GO/idarubicin regimen where the efficacy of the regimen may have overcome the adverse effects of these mutations. Recent studies suggest an adverse impact of epigenetic mutations (*ASXL2* or cohesin/spliceosome mutations). Older patients with CBF AML are treated with lower adjusted dose FLAG-GO/IDA. Patients who cannot tolerate FLAG-GO/IDA or who have persistence molecular MRD positivity may be offered HMA therapy (decitabine, azacitidine) with venetoclax/GO, the treatment duration adjusted according to the MRD results or for 12+ months. Targeted therapies may also be considered (avapritinib or dasatinib for *c-KIT* mutations; FLT3 inhibitors for *FLT3* mutations)^50,51^.

Figure 3 shows the MD Anderson outcomes in CBF AML over the decades.

## Younger patients with acute myeloid leukemia (and/or older patients fit for intensive chemotherapy)

The median age in AML is 68 years^85^. Most of the research with 3 + 7 and other intensive chemotherapy regimens was conducted in younger patients (usual upper age limit 60–65 years). The published results of these trials may not reflect the actual results in the community practice (discussed later)^85^.

### The “3 + 7” anthracyclines-cytarabine regimens; high-dose cytarabine consolidations

The discovery of the anti-AML activity of cytarabine and anthracyclines in the 1970s led to a series of randomized trials evaluating different doses and schedules of cytarabine (5 versus 7 versus 10 days; 100 mg/m^2^ versus 200 mg/m^2^) in combination with anthracyclines, and the addition of other agents (etoposide, 6-mercaptopurine, 6-thioguanine, others) to induction-maintenance therapy. These studies established the 3 + 7 regimen as a standard of care over the next 40 years. The 3 + 7 refers to 3 days of anthracyclines (daunorubicin 30–60 mg/m² intravenously [IV] daily × 3; idarubicin 12 mg/m² IV daily × 3 days) and cytarabine (100–200 mg/m² IV as a continuous infusion daily for 7 days). Consolidation strategies have investigated multiple courses of chemotherapy with cytarabine and anthracyclines, as well as high-dose cytarabine. A randomized trial by Meyer and the Cancer and Acute Leukemia Group B (CALGB) reported significantly superior survival using high-dose cytarabine consolidation therapy (3 g/m² IV over 2–3 h every 12 h on Days 1, 3, and 5) for four courses, compared with lower cytarabine dose schedules^86^. In the CALGB study, high-dose cytarabine consolidations were followed by four additional courses of 2 + 5 chemotherapy. The latter addition was omitted from the subsequent comparative trials, which may be important (later studies using this regimen reported 5-year survival rates of 20–30% rather than 40%)^7^. High-dose cytarabine then became the consolidation standard of care in AML. Other studies investigated lower doses of high-dose cytarabine (1.5 g/m^2^), 4–5 courses versus lower numbers of consolidation courses, and the possible benefits of using allogeneic or autologous SCT in first CR^87^.

### Better regimens than 3 + 7

An increasing body of research suggests that there are better induction-consolidation regimens than 3 + 7. Modifications of frontline AML therapy include: (1) The use high-dose cytarabine combination during induction. (2) Optimization of the dose of daunorubicin (60 mg/m^2^ daily × 3, versus 45 mg/m^2^ or 90 mg/m^2^ daily × 3) and the use of other anthracyclines (idarubicin, mitoxantrone). (3) The addition of adenosine nucleoside analogs (fludarabine, clofarabine, cladribine) to cytarabine-anthracyclines. (4) The addition of the CD33-targeted monoclonal antibody (GO). (5) The addition of targeted therapies such as FLT3 and IDH inhibitors in appropriate patients. (6) The addition of the BCL-2 inhibitor venetoclax to induction therapy on investigational trials. (7) The use of maintenance therapy with oral azacitidine.

### High-dose cytarabine induction

High-dose cytarabine (1–3 g/m^2^ twice daily on Days 1, 3 and 5 or daily × 5) in AML consolidation is an established standard of care^86,88,89^. Several studies evaluated high-dose cytarabine during induction. A SWOG trial randomizing younger patients (<65 years) to standard-dose cytarabine (200 mg/m^2^ daily × 7) versus high-dose cytarabine (2 g/m^2^ every 12 h × 12) during induction (both with daunorubicin) showed a higher 4-year relapse-free survival (RFS) rate with high-dose cytarabine among younger (<50 years; 33% versus 21%) and older patients (50 to 64 years; 21% versus 9%; *p* = 0.049)^88^. An Australian study randomizing 301 younger patients (60 years or less) to high-dose cytarabine (3 g/m^2^ every 12 h × 8) or standard-dose cytarabine (both with daunorubicin and etoposide induction) reported significant improvements in CR duration (median 45 versus 12 months; *p* = 0.0004) and 5-year RFS rate (49% versus 24%) with high-dose cytarabine^89^. A meta-analysis of three trials in 1691 patients randomized to induction therapy with high-dose versus standard- dose cytarabine reported improved 4-year rates of RFS (*p* = 0.03), overall survival (*p* = 0.0005) and EFS (*p* < 0.0001) with high-dose cytarabine^90^.

Lowenberg and colleagues^91^ randomized 858 younger patients (median age 49 years; range 18 to 60 years) to induction therapy with high-dose cytarabine 1 g/m^2^ every 12 h × 10 versus standard-dose cytarabine 200 mg/m^2^ daily × 7, both in combination with idarubicin. They reported similar CR, EFS, and survival rates in the two study arms. This study results may have been confounded by the study design, in which all patients received high-dose cytarabine during induction Course 2 (either 2 g/m^2^ every 12 h × 8—total dose 16 g/m^2^—for patients randomized to high-dose cytarabine during Course 1; or cytarabine 1 g/m^2^ every 12 h × 6 days—total dose 12 g/m^2^—for patients randomized to standard-dose cytarabine during Course 1). Thus, all patients received high- dose cytarabine during the two induction courses.

Willemze and colleagues^92^ (EORTC-GIMEMA) conducted a randomized trial in which 1942 younger patients (60 years or less) received daunorubicin plus etoposide and high-dose cytarabine 3 g/m^2^ every 12 h × 8 versus standard-dose cytarabine 100 mg/m^2^ daily × 10. High-dose cytarabine was associated with significantly higher CR rates (82% versus 76%; *p* = 0.01), 6-year EFS rates (44% versus 35%; *p* = 0.003), and 6-year survival rates (52% versus 43%; *p* = 0.009) among patients 15–45 years old. Among patients 45–60 years, high-dose cytarabine was also associated with significant improvements in CR and 6-year EFS rates, as well as a trend for better survival among patients with *FLT3-*ITD AML or poor prognosis karyotypes.

Bassan and colleagues^93^ randomized 574 patients (median age 52 years; range 16 to 73 years) to ICE (idarubicin-cytarabine-etoposide) or idarubicin plus sequential high-dose cytarabine (2-weekly 3-day blocks of cytarabine 2 g/m^2^ twice daily × 2 days). Sequential high-dose cytarabine induction was associated with a significantly higher CR rate post Course 1(81% versus 69%; *p* = 0.02), and significantly better rates of 5-year survival (49% versus 39%; *p* = 0.045) and RFS (48% versus 36%; *p* = 0.028).

A recent SWOG trial (SWOG-1203) randomized patients to: (1) 3 + 7 induction followed by four consolidations with high-dose cytarabine (3 g/m^2^ twice daily on Days 1, 3, and 5—total cytarabine 18 g/m^2^/course x 4 = 72 g/m^2^), (2) IA regimen:Idarubicin plus continuous high-dose cytarabine (1.5 g/m^2^ continuous infusion daily × 4) followed by IA consolidations with cytarabine 0.75 g/m^2^ continuous infusion daily × 3 days ( = 2.25 g/m^2^/course) × 4 (total cytarabine 15 g/m^2^); (3) IA + vorinostat^94^. While the latter two arms were presumably testing the benefit of high-dose cytarabine induction, the total dose of cytarabine was 4.5 times higher with the 3 + 7 regimen compared with the IA regimen. As expected, the 3 + 7 regimen, delivering more total high-dose cytarabine, was superior in the CBF AML. However, despite the lower total cytarabine dose given in IA, the results of 3 + 7 and IA were similar among patients with intermediate or adverse karyotypes. The design of this trial unfortunately did not allow a real testing of the benefit of high-dose cytarabine added to induction.

### Addition of nucleoside analogs

A combination regimen of fludarabine, high-dose cytarabine and idarubicin combination (FLAG-IDA or FAI), was developed at MD Anderson based the preclinical studies of Plunkett et al.^95–98^. The Medical research Council (MRC) AML 15 randomized trial compared the FLAG-IDA in younger patients with AML to 3 + 7 regimens without or with etoposide. The FLAG-IDA regimen consists of cytarabine 2 g/m^2^ daily for 5 days, fludarabine 30 mg/m^2^ daily for 5 days, and idarubicin 8–10 mg/m^2^ daily for 3 days. Among patients who tolerated four courses on the FLAG-IDA arm (2 FLAG-IDA + 2 high-dose cytarabine), the 8-year survival rate was 66% versus 47% in the standard arm^15,87,98^. The FLAG-IDA/FAI is intensive and difficult to deliver due to side effects related to myelosuppression, but likely not more than allogeneic SCT, and possibly worth a 20% difference in 8-year survival. The FLAG-IDA/FAI is not a simple exploration of high-dose cytarabine, but a multi-faceted strategy (addition of fludarabine, idarubicin instead of daunorubicin, high-dose cytarabine induction) that may be better than 3 + 7 when administered at AML centers of excellence. Improved leukemia management expertise (supportive care; antibiotics and antifungal prophylaxis; timely transfusions support, management of toxicity and treatment of infections/sepsis) would allow safe and full delivery of this regimen.

The optimal dose of high-dose cytarabine is unknown even after 30+ years of research of different high-dose cytarabine schedules. Cytarabine 3 g/m^2^ may be beyond the dose required to maximize the anti-AML effect, and may increase toxicity. High-dose cytarabine 1.5–2 g/m^2^ may be equally effective and less toxic. The MRC studies compared high-dose cytarabine 1.5 g/m^2^ versus 3 g/m^2^ during consolidation, and four versus five courses, reporting equivalent results^87^. A study from Korea showed that high-dose cytarabine 1.5 g/m^2^ or more during consolidation was associated with better RFS and survival rates compared with cytarabine 1 g/m^2^^99^. At MD Anderson, we use high-dose cytarabine 1.5–2 g/m^2^ daily × 5 (total 7.5–10 g/m^2^ per course) during induction and consolidations.

Other adenosine nucleoside analogs (clofarabine, cladribine) have also been explored in combinations with standard chemotherapy.

The Polish investigators added cladribine to frontline 3 + 7 induction chemotherapy in two sequential randomized trials. In the first study, they randomized 400 patients to induction with 3 + 7 + /− cladribine, and reported that adding cladribine produced higher CR (64% versus 46%; *p* = 0.0009) and leukemia-free survival rates (44% versus 28%; *p* = 0.05)^100^. In the subsequent study, they compared three arms, two of them adding cladribine or fludarabine. They showed again that the addition of cladribine (but not fludarabine) resulted in higher CR (67.5% versus 56%; *p* = 0.001) and 3-year survival rates (45% versus 33%; *p* = 0.02)^101^.

At MD Anderson, we continue to use AML regimens that add adenosine nucleoside analogs like fludarabine (FAI, FLAG-IDA regimens), clofarabine (CIA regimen) and cladribine (CLIA regimen) to idarubicin and high-dose cytarabine as frontline induction therapy in younger patients with AML^102^. All patients with *FLT3-*mutated AML now receive gilteritinib or quizartinib during induction and consolidation. Based on the positive experiences of combining FLT3 inhibitors (midostaurin, sorafenib, gilteritinib) with chemotherapy from pilot studies and from the German and Intergroup randomized trials (discussed later), this approach may become a standard of care in *FLT3-* mutated AML, but also perhaps in all patients with AML regardless of *FLT3* mutation status.

### Choice of anthracycline

The better anthracycline and its optimal dose have been the subject of several randomized trials. Historically, daunorubicin 30–60 mg/m^2^ daily × 3 was used for induction therapy. Two studies compared higher-dose daunorubicin 90 mg/m^2^ daily × 3 to daunorubicin 45 mg/m^2^ daily × 3 (in combination with cytarabine) in younger (age <60 years) and older patients (age 60+ years)^6,7^. In younger patients, high-dose daunorubicin was associated with a significantly higher CR rate (71% versus 57%; *p* < 0.001) and longer survival (median 24 versus 16 months; *p* = 0.003). However, the benefit was observed only in patients younger than 50 years and those with normal karyotypes^7^. In older patients, high-dose daunorubicin was associated with a higher CR rate (64% versus 54%; *p* = 0.002) but not with improved survival, although a survival benefit was observed in the subset of patients 60 to 65 years old^6^. Daunorubicin 45 mg/m^2^ daily × 3 is sub-standard. Daunorubicin 60 mg/m^2^ daily × 3 may be as effective and less toxic than 90 mg/m^2^ daily × 3. A French study analyzed 402 patients (median age 49 years) who received daunorubicin 60 mg/m^2^ versus 90 mg/m^2^ as part of 3 + 7 induction, and reported no difference in CR, induction mortality, RFS or overall survival^103^. A MRC study compared daunorubicin 60/m^2^ versus 90 mg/m^2^ during induction and reported no difference in the longer term outcome (2-year survival 60% versus 59%; p 0.14), but a higher early mortality with daunorubicin 90 mg/m^2^ ^104^. These studies helped establish daunorubicin 60 mg/m^2^ daily × 3 as the likely optimal dose schedule of daunorubicin.

Studies comparing idarubicin to daunorubicin, including a meta-analysis of five randomized trials, indicated that idarubicin may be associated with higher CR and survival rates^105^. Pautas and colleagues^106^ randomized 468 patients to induction therapy of standard-dose cytarabine in combination with daunorubicin 80 mg/m^2^ daily × 3 versus idarubicin 12 mg/m^2^ daily × 3 or 4 days. Idarubicin for 3 days resulted in a higher CR rate (83% versus 70%; *p* = 0.007) and a trend for better 4-year EFS (21% versus 12%) and survival (32% versus 23%) rates. A four-day schedule of idarubicin was not better. A retrospective analysis of two large French trials comparing idarubicin to daunorubicin in 727 patients reported that idarubicin 12 mg/m^2^ daily for 3 days resulted in significantly higher CR (69% versus 61%; *p* = 0.03) and cure rates (16.6% versus 9.8%; *p* = 0.018) compared with daunorubicin^107^. Mandelli and the Italian colleagues^108^ randomized 2157 patients to daunorubicin (50 mg/m^2^ daily × 3), idarubicin (10 mg/m^2^ daily × 3), or mitroxantrone (12 mg/m^2^ daily × 3), in combination with standard-dose cytarabine. Both idarubicin and mitroxantrone were associated with higher 5-year RFS (37% versus 29%; *p* = 0.02) and survival rates (43% versus 45% versus 36%; *p* = 0.01) among patients who did not receive allogeneic SCT. At MD Anderson, we use idarubicin 8–10 mg/m^2^ daily × 3 as part of the FAI/CLIA AML frontline regimens.

### Gemtuzumab ozogamicin

Antibody-targeting therapy is a major success story in hematologic malignancies, particularly in lymphoid malignancies (antibodies targeting CD20, CD19, and CD22 in lymphomas, chronic lymphocytic leukemia, acute lymphoblastic leukemia). The development of GO, a CD33 monoclonal antibody bound to calicheamicin, has had a rough journey in AML. The Food and Drug Administration (FDA) originally granted accelerated approval of GO (9 mg/m^2^ on Days 1 and 15) in the US in May 2000 for the treatment of older patients (60 years or older) in first relapse who are not candidates for cytotoxic chemotherapy. This approval was based on three phase 2 studies in 142 patients with relapsed AML (response rate 30%; CR rate 16%)^109^. The approval was conditional on a future demonstration of the GO benefit in randomized trials. Several studies then explored lower and fractionated dose GO schedules in frontline randomized trials (3 mg/m^2^ × 1 during induction and consolidation; 3 mg/m^2^ on Days 1, 4, and 7 during induction). The pivotal trial in the US^16^ randomized patients to standard 3 + 7 with daunorubicin 60 mg/m^2^ daily × 3, versus the addition of GO 6 mg/m^2^ on Day 4 to 3 + 7, but with daunorubicin 45 mg/m^2^ daily × 3 (equitoxic but suboptimal dose in retrospect). They reported a higher induction mortality rate with GO (5% versus 1%), which resulted in the withdrawal of GO from the US market in 2010^16^. This study had an unusually low mortality rate in the standard arm (usually about 3–7%), which suggested that GO may have increased mortality. The dose of daunorubicin in the GO arm was suboptimal, as confirmed today by several studies (discussed earlier). Four other randomized trials later matured, all demonstrating the benefit of adding GO, either overall or in subsets of patients^15,98,110,111^. A meta-analysis of the five randomized trials involving 3,325 patients showed that the addition of GO did not increase the CR rate, reduced the risk of relapse (*p* = 0.0001), and improved the 5-year survival rate (*p* = 0.01). The GO effect was most pronounced in AML with favorable cytogenetics (increased 5-year survival rate from 50 to 75%; *p* = 0.0006) and intermediate cytogenetics (*p* = 0.005). Gemtuzumab 3 mg/m^2^ was associated with fewer early deaths than 6 mg/m^2^ and provided equal efficacy^17^. This resulted in the FDA re-approval of GO at the lower dose schedules for AML therapy in 2017^112,113^.

### French experience with lomustine in older AML on 3 + 7

In three French studies involving 847 older patients (>60 years), the investigators reported that the addition of lomustine (alkylating agent) 200 mg/m^2^ orally on Day 1 to idarubicin + cytarabine (*n* = 508), compared with idarubicin + cytarabine (*n* = 339), was associated with a higher CR rate (68% versus 58%; *p* = 0.002), a similar rate of toxic deaths, and a longer survival (median 12.7 versus 8.7 months; *p* = 0.004). By multivariate analysis, lomustine was an independent favorable treatment variable for achievement of CR (*p* = 0.002) and for survival prolongation (*p* = 0.002)^114^.

### The MD Anderson approach in 2020

To summarize, the optimal frontline therapy for younger patients with AML is evolving. While many AML experts (and community oncologists) favor 3 + 7 as the standard of care, better regimens may have emerged. These incorporate high-dose cytarabine during induction and consolidations, include nucleoside analogs into the regimens, may incorporate lower-dose GO as part of induction-consolidation in CBF and intermediate-karyotype AML, may add other targeted therapies, particularly FLT3 inhibitors (e.g., gilteritinib, midostaurin, sorafenib) in *FLT3*-mutated AML, and may add venetoclax to regimens in non *FLT3-* mutated AML (discussed later).

**Fig. 4:**
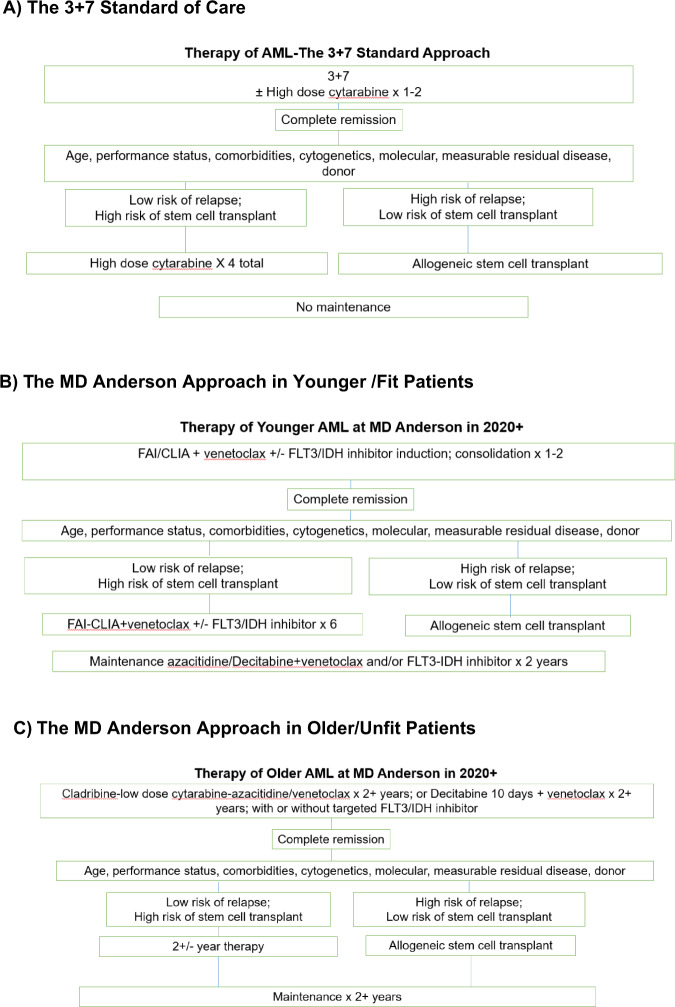
Therapy of AML. **A** standard of care; **B** MD Anderson approach in young/fit patients, and **C** in older patients.

AT MD Anderson, younger patients with AML referred today are treated with a combination of idarubicin, high-dose cytarabine and an adenosine nucleoside analog (fludarabine—FAI/FLAG-IDA; cladribine—CLIA). FLT3 inhibitors (gilteritinib, quizartinib) are added to the regimen in patients with *FLT3-*mutated AML. Venetoclax shorter courses (7–14 days) are under investigation in combination with FAI or CLIA in the other AML subsets^115,116^. Once in CR, and based on availability of donors, patient age and co-morbidities, pretreatment AML characteristics (cytogenetics, molecular profiles) and MRD status in CR, patients may be offered allogeneic SCT. On average, patients are considered for allogeneic SCT in first CR if they have high-risk disease based on adverse cytogenetic abnormalities, high *FLT3-*mutation AR, or persistent MRD >0.1% in CR post first consolidation. Otherwise, they complete 4–6 courses of consolidation and are then offered maintenance therapy with azacitidine and venetoclax for 2+years, with or without the addition of targeted inhibitors (e.g., FLT3 inhibitors if *FLT3*-mutated AML; IDH inhibitors if *IDH*-mutated AML). Figure 4 shows the approaches in community practice and at MD Anderson. Patients 50 years or older are offered induction therapy in the protected environment to reduce induction mortality (Table 4). In community practice, reasonable isolation procedures could be proposed: laminar air-flow rooms; reverse isolation; gloves, masks, gowns; no plants or flowers; limiting visitors. Intensive supportive care is offered with antibiotic prophylaxis including antifungals (posaconazole or voriconazole)^117,118^.

**Table 4:** General approach to patients with AML at MD Anderson in 2020.

Disease	Therapy and comments	% 5-year survival
APL	-ATRA plus arsenic trioxide- -GO added for high-risk APL or persistent molecular disease ≥ 2–3 months into CR	80–90
CBF AML	-FLAG-GO induction + 4 + 6 consolidations -age ≥ 60 years: adjusted dose FLAG-GO -Intolerance to FLAG-GO: decitabine or azacitidine × 12 (according to molecular MRD) ± targeted therapies (i.e., GO as tolerated); -Monitor response with real-time qPCR testing (goal: >3-log reduction)	80
AML in younger patients	-FLAG-IDA, CLIA induction + 6 consolidations -FLT3 ITD: add FLT3-inhibitor (gilteritinib on study) -Clinical trials: venetoclax added to CLIA or FIA; -Future: activity of FLT3 inhibitors regardless of *FLT3* status; IDH inhibitors + chemotherapy in patients with *IDH1/2* mutations	40–50
AML in older patients/unfit for intensive chemotherapy (age > 60–70 years; 8-week mortality ≥ 20–30%)	-Cladribine plus low-dose cytarabine alternating with HMA -Clinical trial: cladribine-low-dose cytarabine- azacitidine + venetoclax -Clinical trial: decitabine 10 days + venetoclax -“Triplet” combinations on clinical trials (mutation specific): Decitabine/azacitidine + venetoclax + quizartinib/gilteritinib (*FLT3*-mutated) Azacitidine + venetoclax + IDH inhibitor (IDH mutated) Azacitidine + venetoclax + APR246 (TP53 mutated) Azacitidine + venetoclax + magrolimab -Other investigational agents	20–30
Allogeneic SCT	-In CR1 if poor cytogenetics, or FLT3 ITD high allelic ratio, or adverse mutations, or MRD positive in CR, and low treatment related mortality of SCT procedure -CR2 and beyond: all potential patients	
Salvage therapy	-CRD1 ≥ 12 months: high-dose cytarabine-based regimens -FLAG-IDA + Venetoclax on clinical trial -CRD1 < 12 months: phase 1–2 trials -Always recheck for mutations (next-generation sequencing), particularly for *FLT3* and *IDH1/2* mutations; if mutations then target-based therapy	
Supportive measures	-Antibiotic/antifungal prophylaxis -Protected environment/reverse isolation if age ≥ 50 years + intensive chemotherapy, or if age ≥ 60 years + low-intensity therapy	

*APL* acute promyelocytic leukemia, *ATRA* all-transretinoic acid, *GO* gemtuzumab ozogamicin, *FLAG-Ida*, FAI fludarabine, high-dose cytarabine, idarubicin, *CLIA* cladribine, high-dose cytarabine, idarubicin, *HMA* hypomethylating agent.

With this general approach, the CR rate among non-selected younger patients with AML is 70–80%, and the long-term survival rate is 40–50% (Fig. 1). With the encouraging data incorporating venetoclax, FLT3 inhibitors, IDH inhibitors, and monoclonal antibodies (GO, novel CD33 monoclonal antibodies), combined modality strategies involving targeted agents and chemotherapy are becoming a reality in the management of all younger and older patients with AML.

Since the 2015 AML review^1^, many of the strategies listed then as investigational are now FDA approved and used as standards of care, either in the FDA approved indications, or in combined modality therapies that synergize their clinical benefits and render them more cost-effective. This is certainly the case for venetoclax, FLT3 inhibitors (gilteritinib), and IDH inhibitors (enasidenib, ivosidenib). Next, we summarize such ongoing studies with intensive chemotherapy in younger patients with AML.

### Regimens with venetoclax

At MD Anderson, the frontline regimens, FLAG/IDA and CLIA, are now combined with venetoclax for 7–14 days during induction and for 5–7 days in maintenance, as tolerated^115^. The preliminary data are encouraging. Among 28 patients treated with FLAG/idarubicin-venetoclax, the overall response rate is 93%, and the MRD negativity rate in CR 92%^115^. Among 31 patients treated with CLIA-venetoclax, the overall response rate is 90%, and the estimated 1-year survival 78%^116^. The regimens are myelosuppressive as expected, but tolerable with very low rates of induction mortality. Growth factors and prophylactic antibiotics/antifungals are essential to reduce the risk and morbidity of opportunistic infections.

### Regimens with IDH inhibitors

Stein and colleagues^119^ treated 134 patients with de novo AML and IDH mutations using a combination of 3 + 7 and ivosidenib (IDH1 mutation; *n* = 60) or enasidenib (IDH2 mutation; *n* = 91). With 3 + 7 + ivosidenib the overall response rate was 93% and the estimated 1-year survival rate 79%. With 3 + 7 + enasidenib the overall response rate was 73% and the estimated 1-year survival rate 75%^119^. The HOVON and German study groups are currently evaluating 7 + 3 with either ivosidenib or enasidenib (versus placebo) in a large Phase III randomized study (NCT03839771).

### Regimens with FLT3 inhibitors

Stone and colleagues^120^ conducted a randomized phase III RATIFY trial (CALGB 10603) in 717 patients <60 years of age with newly diagnosed *FLT3*-mutated AML (*FLT3-*ITD and/or *FLT3-*TKD; median age 48 years; range 18 to 60 years) with the combination of 3 + 7 with or without midostaurin. Seventy-seven percent of patients had a *FLT3-*ITD mutation and 23% had a *FLT3-*TKD mutation. The addition of midostaurin improved the CR rate (59% versus 54%, *p* = 0.045) and the survival (median survival 74.7 versus 25.6 months, *p* = 0.009; estimated 5-year survival rate 50% versus 42%). The benefit was noted in *FLT3-*ITD low AR (AR less or equal 0.70), *FLT3-*ITD high AR (AR >0.70) and TKD*-*mutated AML. At MD Anderson, a matched-cohort analysis similarly showed the benefit of adding sorafenib to idarubicin-cytarabine in *FLT3*-mutated AML^41^. In our study of CLIA + FLT3 inhibitor (sorafenib/midostaurin), the CR rate was 86% and the estimated 1-year survival 70%^121^.

Several studies are now underway evaluating newer generation FLT3 inhibitors (gilteritinib, quizartinib, crenolanib) in combination with intensive chemotherapy. Pratz and colleagues^122^ treated 33 patients with newly diagnosed AML with 3 + 7 plus gilteritinib, reporting a marrow CR rate of 80+% and an estimated 2-year survival rate of 70%. These encouraging data have led to two randomized studies of 3 + 7 + gilteritinib versus 3 + 7 + midostaurin in Europe (HOVON 156ML; NCT04027309) and the US (NCT03836209). A phase III, randomized study of 3 + 7 + quizartinib versus 3 + 7 in frontline *FLT3-*ITD AML completed enrollment; the results are expected in 2021 (QUANTUM-First, NCT02668653)

Sorafenib has been used as maintenance therapy post allogeneic SCT in *FLT3*-mutated AML in single arm and randomized trials, all showing survival and/or RFS benefits for the addition of sorafenib maintenance^123,124^. A randomized study of gilteritinib versus placebo administered after allogeneic SCT in *FLT3*-mutated AML may help address more definitively the benefit and optimal use of FLT3 inhibitors in this setting. (BMT CTN 1506; ClinicalTrials.gov identifier: NCT02997202).

While FLT3 inhibitors are now established therapies in combination regimens in *FLT3*-mutated AML, it is of interest that several non-targeted chemotherapy strategies have also shown selective benefits in *FLT3*-mutated AML, including induction regimens containing high-dose cytarabine, cladribine and high-dose daunorubicin^92,125,126^.

## Older patients with acute myeloid leukemia (or younger patients not fit for intensive chemotherapy)

### Intensive chemotherapy

The median age of patients with AML is 68 years, but most of the experience with 3 + 7 and intensive chemotherapy regimens is in younger patients, usually 60 years or younger. Older patients with AML tolerate intensive chemotherapy poorly. In the study by Lowenberg and colleagues^6^ evaluating 3 + 7 with daunorubicin 45 mg/m^2^ versus 90 mg/m^2^ daily × 3, among 813 selected patients 60 years and older (median age 67 years), the median survival was 7 to 8 months and the estimated 3-year survival rate was 20%. The study reported an acceptable low early mortality rate of 11–12%. Whether this mortality rate is replicable in unselected patients in oncology community practice is questionable.

The treatment of older patients with AML remains challenging. Acute myeloid leukemia in older patients carries a distinctly different disease biology associated with high risk and often complex karyotype, a high incidence of cytogenetic abnormalities involving monosomies 5 and 7 and chromosome 17 abnormalities, a high incidence of multiple mutations including *TP53* (20+%), and a high incidence of secondary/therapy-related AML (20 to 30%). Older patients have multiple co-morbidities (hypertension; diabetes; organ dysfunctions including cardiac, pulmonary and renal abnormalities) that result in poor tolerance to intensive chemotherapy and high early (4- to 8-week) mortality rates. In community practice (SEER data; 2010–2017) treating unselected older patients, the 4-week mortality is 24% among patients 60–69 years old and the 5-year survival 18%. Among patients 70 years and older (45% of all AML), the 4-week mortality is 44% and the 5-year survival 4%. Clearly neither intensive chemotherapy nor supportive/hospice care are acceptable options in older AML.

At MD Anderson, historical studies using intensive chemotherapy in older patients with AML (age 60–65 years or older) showed CR rates of 40–50%, 4–8-week mortality rates of 26–36%, median survivals of 4–6 months, and one -year survival rates of <30%^127,128^. By multivariate analysis, independent adverse factors predictive of early mortality with intensive chemotherapy were: age 75 years and older; adverse karyotype with three or more chromosomal abnormalities; presence of an antecedent hematologic disorder; poor performance status (ECOG 2–4); creatinine level 1.3 mg/dl or higher; and treatment outside a protected environment. The expected 8-week mortality was 10–19% with the presence of 0–1 adverse factors, and 36–65% with the presence of 2–5 adverse factors^127^.

### Epigenetic and low-intensity therapy

Faced with the poor results with intensive chemotherapy, investigators began in the 1990’s evaluating lower-intensity strategies in patients unfit for intensive chemotherapy (expected high-early mortality). These included low-dose cytarabine, HMA therapy, and targeted therapies (monoclonal antibodies; more recently FLT3 inhibitors and IDH 1/2 inhibitors). This raised the question of how to select patients unfit for intensive chemotherapy. At our institution, we use the above model to select such patients, based on an estimated early mortality rate in excess of 10%. Over time and over several investigational studies since 2000, we have shown that lower-dose chemotherapy/HMA therapy combinations now provide, since 2015, overall response rates as high as with intensive chemotherapy, significantly lower rates of early mortality and myelosuppression-associated complications, and survival equivalent or superior to intensive chemotherapy^129,130^.

In clinical practice, leukemia experts often base the decision of intensive versus low-intensity therapy on the “oculometer” (looking at the patient and deciding by intuition and experience). The approach is subjective and based on the oncologist’s experience and perception of the patient’s condition (performance, co-morbidities, infections at presentation, tolerance to intensive chemotherapy). This may be better replaced by more objective prediction models such as the one used at our institution. Patients are then categorized according to their predicted early mortality (based on the multivariate prognostic models)^127,128^. If the expected 4–8-week mortality is <10%, they are offered intensive chemotherapy. If it is >10–20%, they are offered low-intensity approaches. Of interest, a third of patients who present as afebrile with normal chest radiographs may have significant abnormalities detected by computerized tomography (CT) scans (infections, nodular lesions suggestive of early fungal pneumonia, bleeding, other)^131^. Patients with AML and pneumonia at diagnosis have a significantly higher risk of early mortality with intensive chemotherapy (4–8-week mortality 15–30%; Sasaki-unpublished). Future studies should investigate incorporating pretreatment routine CT of chest findings into predictive models of early mortality in AML.

Historically, many older patients (age 70 or older) with AML were offered supportive palliative or hospice care^132^. The MRC AML14 study randomized 217 older patients to low-dose cytarabine 20 mg subcutaneously twice daily × 10 days versus supportive care and hydroxurea^133^. Low-dose cytarabine was associated with a higher CR rate (18% versus 1%; *p* = 0.00006) and with longer survival (odds ratio: 0.60; *p* = 0.0009). This study drove home an important message: that an active tolerable treatment would have a significant effect on improving early mortality and overall survival, even among patients deemed suitable only for supportive care at the time of diagnosis. In the 2000s, studies with HMAs demonstrated the benefits of decitabine and azacitidine for the treatment of older patients unfit for intensive chemotherapy. A phase 3 study randomized 485 patients 65 years or older to decitabine 20 mg/m^2^ IV daily × 5 every month versus supportive care or low-dose cytarabine. In a final analysis, the median survival was 7.7 with decitabine versus 5 months with supportive care or low-dose cytarabine (*p* = 0.036). This led to the European Medicines Agency (EMA) approval of decitabine for the treatment of older patients with AML^26^. A similar study (AZA-AML-001) randomized 488 older patients to azacitidine (*n* = 241) versus three predetermined conventional care regimens (*n* = 247; low-dose cytarabine, intensive chemotherapy, supportive care). Azacitidine therapy was associated with longer survival (median 10.4 versus 6.5 months; *p* = 0.06; hazard ratio 0.85)^28^.

Studies have also evaluated longer durations of decitabine schedules (20 mg/m^2^ daily × 10)^134^ in combinations (venetoclax, FLT3 and IDH inhibitors, others). The FDA approved recently a 100% absorbable oral formulation of decitabine plus oral cedazuridine (cytosine deaminase inhibitor; oral combination bioequivalent to intravenous decitabine)^135,136^. This opens research into potentially highly effective oral therapies in older AML (oral decitabine-cedazuridine plus venetoclax), which may improve tolerance and quality of life, and offer safe and effective outpatient therapy.

At MD Anderson, prior to the discovery of the role of venetoclax in AML, we had evaluated sequential three-drug low-intensity therapy combining an adenosine nucleoside analog (clofarabine or cladribine) with low-dose cytarabine, and alternating this with decitabine over a period of 18 months^137,138^. Among 248 patients (median age 69; range 48–85 years) treated with the two regimens, the overall response rate was 66%, the CR rate 59%, the early (4-week) mortality rate 2%, the median survival 12.5 months, and the estimated 2-year survival rate 29%. Among patients with normal karyotype, the median survival was 19.9 months and the estimated 2-year survival rate 45%^137,138^. At that time, compared to single-agent HMAs, which were standard therapy, the triple-nucleoside analog (cladribine-cytarabine-HMA) low-intensity therapy showed better results. It also represented a novel, well-tolerated, effective new backbone therapy upon which to build combination approaches. Deriving from the success of HMA + venetoclax combinations, we are evaluating the combination of cladribine-low-dose cytarabine-azacitidine with venetoclax in older AML.

### Regimens with hypomethylating agents and venetoclax (ABT-199)

One therapeutic strategy to target AML involves activation of the intrinsic or mitochondrial pathway of apoptosis. This pathway is regulated by the BCL2-family of proteins. It involves a dynamic balance of pro-apoptotic effectors (Bak, Bax) and anti-apoptotic proteins (BCL-2, BCL-XL, MCL-1). In a balanced state, the anti-apoptotic proteins bind to and sequester the pro-apoptotic proteins and prevent them from triggering apoptosis. Anti-apoptotic proteins are overexpressed in many tumors including AML. Small molecule “BH3 –mimetics” were developed that bind to the anti-apoptotic proteins in the BH3 domain and liberate pro-apoptotic proteins that subsequently trigger apoptosis. The earlier generation of BH3 mimetics bound efficiently to multiple anti-apoptotic proteins, including BCL-2, BCL-XL, and MCL-1, and thus were associated with unacceptable on- target toxicities, including thrombocytopenia.

Venetoclax (ABT-199; BCL2 inhibitor) was developed over many years as a more advanced BH3 mimetic molecule designed to retain specificity for BCL-2, but without affinity for BCL-XL or MCL-1. Venetoclax has already revolutionized the treatment of chronic lymphocytic leukemia and may have a role in other cancers (acute lymphoblastic leukemia, myelodysplastic syndrome, lymphoma and myeloma subsets). The AML blasts and AML stem cells depend on BCL-2 for survival, but normal hematopoietic stem cells depend on MCL-1. This presented the rationale for investigating venetoclax in AML. Preclinical studies confirmed its activity in AML cell lines, in murine primary xenografts, and in AML samples^139^. A phase 2 single-agent study in AML investigated venetoclax (800 mg daily) in 32 patients with refractory-relapsed AML. The overall response rate was 15%, with another 19% of patients having reductions of blasts^140^. Responses appeared to be more frequent among patients with *IDH* mutations, a clinical observation that confirms preclinical studies, suggesting BCL-2 to be a synthetic lethal partner AML with *IDH1/2* mutations^53,140^.

Based on the encouraging preclinical data of venetoclax in combination with HMAs and low-dose cytarabine, single-arm trials evaluated these combinations in newly diagnosed patients with AML who were older than 75 years or unfit to receive intensive chemotherapy. The positive results (overall response rates 67%; estimated median survival 17.5 months; 2-year survival rate 40%) led to the FDA accelerated approval of venetoclax in combination with epigenetic therapy or low-dose cytarabine for the treatment of these patients^141,142^.

The subsequent VIALE-A pivotal trial randomized such patients (75 + years; or unfit for intensive chemotherapy) to therapy with azacitidine alone or in combination with venetoclax. Among 431 patients randomized on a 2:1 basis to azacitidine plus venetoclax (*n* = 286) or azacitidine (*n* = 145), the addition of venetoclax resulted in a significantly longer survival (median survival 14.7 versus 9.6 months; *p* < 0.001). The overall response rate (66.4% versus 28.3%; *p* < 0.001) and CR rate (29.7% versus 17.9%; *p* < 0.001) were also higher^143^. A similar randomized study (211 patients; 2:1 randomization) of low-dose cytarabine with venetoclax versus low-dose cytarabine alone showed a median survival of 8.4 versus 4.1 months (*p* = 0.04), an overall response rate of 48% versus 13% (*p* < 0.001), and a CR rate of 27% versus 7% (*p* < 0.001)^144^.

A single-arm trial from our institution investigated the use of decitabine for a 10-day induction with venetoclax (followed by maintenance with monthly decitabine for 5 days and venetoclax for 14–21 days). Among 70 older patients (median age 72 years; range 70–78 years) with newly diagnosed de novo AML treated with the regimen, the overall response rate (CR + CRi) was 84%, the CR rate 67%, the 4-week mortality rate 0%, and the median survival 18.1 months^145^.

### Experience with low-intensity chemotherapy combination and venetoclax

One of the current frontline trials in older AML at MD Anderson explores the combination of cladribine-cytarabine-venetoclax alternating with azacitidine-venetoclax^146^. Among 48 patients treated so far (median age 68 years; range 57–84 years), the CR rate was 77%, the overall response rate 94%, the MRD negativity rate 80%, the 4-week mortality rate 0%, and the estimated 1-year survival rate 70%.

### Hypomethylating agents with FLT3 inhibitors

The combination of azacitidine and sorafenib in older patients with FLT3-ITD AML resulted in a CR-CRi rate of 78% and a median survival of 8.3 months^147^.

Gilteritinib was combined with azacitidine in the frontline setting in the ongoing phase III LACEWING trial. The initial safety run-in data from this study showed an encouraging marrow CR rate of 67% among the first 15 patients treated prior to the beginning of the randomization^148^. A gilteritinib dose of 120 mg daily was selected with standard-dose azacitidine (NCT02752035).

Older patients (age 65 years or older) and patients not fit for intensive chemotherapy (based on predicted high early mortality) are now offered low-intensity strategies using combinations of cladribine and low-dose cytarabine alternating with decitabine or azacitidine together with venetoclax; decitabine (10-day induction, 5-day maintenance) combined with venetoclax, and other HMAs (e.g., oral decitabine) plus venetoclax-based combinations that also incorporate FLT3 inhibitors (if *FLT3*-mutated AML), IDH inhibitors (if *IDH*-mutated AML), or APR246 or magrolimab (if *TP53*-mutated AML). (Table 4).

### CPX-351 in older AML

CPX-351 is a nano-scale liposome, which contains a fixed 5:1 molar ratio of cytarabine and daunorubicin^149^. Following the encouraging preclinical and phase 1–2 trials in the subset of secondary AML,a pivotal phase 3 randomized trial in newly diagnosed secondary AML accrued 309 patients randomized to CPX-351 versus 3 + 7. Therapy with CPX-351 was associated with a significantly longer survival (hazard ratio 0.69; *p* = 0.005). The CR rate was 38% with CPX-351 versus 26% with 3 + 7 (*p* = 0.035); the CR + CRi rate was 48% versus 33% (*p* = 0.016). CPX-351 was associated with a longer duration of myelosuppression. More of the patients achieving CR post CPX-351 were able to undergo later allogeneic SCT (20% versus 12%); their survival was also longer post SCT. The study findings resulted in the FDA approval of CPX-351 as frontline therapy of secondary AML^150,151^. Ongoing studies are combining CPX-351 with venetoclax, GO, and other targeted therapies.

### Glasdegib

The Hedgehog (Hh) signaling pathway plays critical roles in embryogenesis and stem cell maintenance. Dysregulations in the Hh pathway can result in the development, maintenance and expansion of the leukemic stem cells, which play a critical role in AML pathogenesis, persistence and progression^152^.

Glasdegib, a cyclopamine derivative, is a selective inhibitor of Smoothened (SMO), a component of the Hh signaling pathway. Following encouraging preclinical and phase 1–2 trials, a phase 2 study investigated low-dose cytarabine alone versus low-dose cytarabine plus glasdegib 100 mg daily. The addition of glasdegib was associated with a significant prolongation of survival (median survival 8.8 months versus 4.9 months; 12-month survival 59.8% versus 38.2%)^153^. This led to the approval of glasdegib for the treatment of newly diagnosed AML in patients 75+ years old or unsuitable for intensive induction chemotherapy^154^. Ongoing studies are evaluating glasdegib combination with azacitidine and with intensive chemotherapy.

### The potential roles of APR-246 and magrolimab in *TP53*-mutated AML

*TP53*- mutated AML is associated with older age, therapy-related disease, complex (adverse) cytogenetics, and very poor prognosis. Even with the advent of HMAs plus venetoclax, older patients with *TP53*-mutated AML ineligible for induction therapy continue to do poorly: response rates 50% but median survival only 3–6 months^145^.

APR-246 is a novel agent that may restore the transcriptional activity of unfolded wild-type or mutant *p53*, leading to induction of apoptosis in cancer cells with mutant *p53*^155^. In two parallel ongoing studies in France and the US, the combination of azacitidine with APR-246 produced CR/CRi rates of 60–80%; >60% of responders had undetectable *TP53* mutation by next-generation sequencing^156,157^. A phase III randomized study of azacitidine with or without APR-246 in frontline MDS and AML with 20 to 30% blasts has recently completed enrollment (NCT03745716), and reported not to have met the study primary endpoint of significantly higher CR rate (December 28, 2020).

CD47 functions as a macrophage checkpoint, providing a potent “do not eat me” signal that allows tumor cell evasion and immune destruction by macrophages. CD47 is upregulated in AML, and CD47 upregulation was independently associated with a poor prognosis^158–160^. Hu5F9-G4 (magrolimab) is a humanized monoclonal antibody that binds CD47 and blocks it from interacting with its ligand SIRPα on phagocytic cells, leading to phagocytic elimination of cancer cells. The combination of magrolimab plus azacitidine was evaluated in patients with newly diagnosed AML who were unfit for intensive chemotherapy or who had MDS intermediate-higher risk (Revised International Prognostic Scoring System [IPSS-R]). In 34 evaluable patients with AML, the objective response rate was 65%, (CR 40%, CRi 12%). The median time to response was 2.0 months. Among patients who had abnormal cytogenetics at baseline, 47% achieved complete cytogenetic response. In patients harboring *TP53* mutations, the overall response rate was 71% (15 of 21 patients) and the CR rate 48 % (5 of 12 patients). The median survival in *TP53*-mutant AML was 12.9 months and in TP53 wild-type 18.9 months^161^.

## Maintenance therapy in acute myeloid leukemia

Maintenance therapy is an established positive approach in many cancers, including acute lymphocytic leukemia. However, studies in AML could not confirm a clear benefit of maintenance therapy, until the recent positive results reported with oral azacitidine (CC-486). The oral drug is poorly absorbed (AUC 10–30% of intravenous azacitidine). In an international multi-center trial (QUAZAR AML-001), 472 patients 55 years and older (median age 68 years) with AML in first CR for <4 months were randomized to oral azacitidine (CC-486) 300 mg orally daily × 14 every month (*n* = 238), or placebo (*n* = 234). The median survival was 24.7 months with CC486 versus 14.8 months with placebo (hazard ratio 0.69, *p* = 0.0009). The median RFSs were 10.2 and 4.8 months. The FDA approved CC-486 as oral maintenance therapy for this indication in September 2020^162^.

A second study (HOVON97) randomized 116 patients 60 years and older with AML who were in CR post two courses of intensive chemotherapy to azacitidine 50 mg/m^2^ subcutaneously daily × 5 every month for 12 courses (*n* = 56) versus observation (*n* = 60). The 12-month disease-free survival (DFS) was 64% with azacitidine versus 42% with observation (*p* = 0.04)^163^

In the context of post-SCT, maintenance therapy has also been of benefit. Buchert and colleagues^124^ reported on 83 patients (median age 54 years) with *FLT3-*ITD AML post allogeneic SCT who were randomized to sorafenib 200–400 mg twice daily for two years versus placebo. The 2-year PFS rate was 85% with sorafenib versus 53% with placebo (*p* = 0.04). Survival was also better (hazard ratio 0.447; *p* = 0.03). In the pivotal gilteritinib versus salvage chemotherapy (ADMIRAL) trial in 371 patients with *FLT3-*mutated AML, Perl and colleagues^164^ reported on 51 patients achieving a response post gilteritinib and undergoing allogeneic SCT who either resumed gilteritinib post SCT (*n* = 35) or did not (*n* = 16). The median survival was longer with gilteritinib resumption (16.2 months versus 8.4 months; hazard ratio 0.387; *p* = 0.024).

## Translating the published literature into real-world experience

Here a word of caution—an analysis of the SEER data (more reflective of the reality on the ground and of general oncology community practice) in about 29,000 patients with AML showed results substantially worse than those reported from single institutions and from cooperative trials. In the SEER data, the results have improved since 2000 in APL (5-year survival about 60 + %) and CBF AML (5-year survival 50%), mostly in patients younger than 60 years. However, even restricting the analysis to the Years 2000–2017, the 4-week mortality among patients 40–59 years old with de novo AML (excluding APL and CBF AML) is 27% and the 5-year survival rate 40%. Among patients 70+ years old, the 4-week mortality rate is 45–50% and the 5-year survival rate <5%^85^.

Therapy of AML is difficult and requires long-term expertise. This is because AML is rare, and often affects older patients who require chemotherapy in the setting of a compromised marrow by the disease; this results in severe cytopenias at diagnosis and throughout therapy. All these conditions require also the use of antibiotics prophylaxis and the prompt availability of optimal supportive care (platelets and blood transfusions; skilled emergency centers and facilities to deliver the support needed, recognize infections and sepsis, implement proper broad-spectrum IV antibiotics, and offer timely intensive care unit care when needed). Thus, the risks of serious morbidities, mortality and treatment abandonment are high.

For a long time, it was assumed that AML care may be equally optimal in the community practice as it is in published data from cooperative trials. This, however, may not be the case. In several AML cooperative trials, the early (4-week) mortality with intensive chemotherapy in younger patients with AML ranges from 1 to 10%^91,165^. At our institution, the early mortality with intensive chemotherapy is <5%; the early mortality with low-intensity regimens in older AML is 1–2%.

Two recent studies reported significantly higher early mortality rates among patients treated in non-academic versus academic centers, and in non-NCI-designated versus NCI-designated cancer centers^166,167^. In a National Cancer data Base of 60,738 patients with AML, the 1-month mortality was 16% in academic centers and 29% in non-academic centers (*p* < 0.001), and the 5-year survival rate was 25% versus 15 % (*p* < 0.001)^166,167^. The second study from California in 7007 patients with AML reported an early mortality rate in AML of 12% in NCI-designated cancer centers versus 24% in non-NCI-designated cancer center^167^.

Perhaps AML, being rare and requiring intensive chemotherapy and supportive care in the setting of a compromised marrow, is better treated in specialized leukemia centers, rather than in the community practice.

## Allogeneic and autologous stem cell transplantation

A meta-analysis combining data of multiple randomized trials demonstrated the significant benefit, on average, of allogeneic SCT in first CR^168^. The value of allogeneic SCT in AML first CR was difficult to confirm in earlier randomized trials because of: (1) the limited number of patients in each study (may not detect modest but clinically significant benefits); (2) the lead time bias to allogeneic SCT; (3) many patients allocated to allogeneic SCT could not undergo the SCT (infections, organ dysfunction, new chemotherapy related morbidities, AML relapse, others); (4) patients allocated to chemotherapy in first CR may have benefited from an allogeneic SCT in second CR. A study by the MRC reported that the benefits of chemotherapy versus allogeneic SCT in first CR were similar when the benefit of allogeneic SCT in second CR was factored in^169^.

Allogeneic SCT is an accepted standard of care in first CR, and based on several patient, AML and treatment-associated factors: (1) the presence of an adverse AML karyotype or high *FLT3-*mutated AR at diagnosis; (2) persistent MRD in CR; (3) low-risk of SCT-associated mortality based on the patient’s age and co-morbidities, donor availability and degree of matching. With the FDA approval and availability of venetoclax and FLT3 and IDH inhibitors, the role of allogeneic SCT in first CR needs to be continuously evaluated.

Allogeneic SCT should not be considered as a one-time independent procedure, but part of the total strategy of chemotherapy-targeted therapy-SCT. Investigations of post allogeneic SCTmaintenance strategy to reduce the risk of relapse should be incorporated into this continuum, including azacitidine-decitabine (parenteral and oral), FLT3 inhibitors, IDH inhibitors, venetoclax, and others.

Autologous SCT has been largely abandoned in the United States because of the lack of a definite benefit. European AML experts still advocate for its role in first CR based on randomized trials showing that autologous SCT provides equivalent results to multiple chemotherapy consolidations (usually fewer than 4). With the knowledge concerning persistence of MRD in CR, it is possible that historical studies may have reinfused autologous marrows with significant persistent AML disease burden, thus perhaps increasing the relapse rates. This may have abrogated the potential benefit of this approach. Future studies may evaluate again the benefit of autologous SCT using collected MRD-negative marrows. At our institution, autologous SCT is still considered occasionally in the setting of APL and CBF AML in second CR and with negative molecular MRD in collected stem cells.

## Salvage therapy

The choice of salvage therapy in AML depends on multiple factors: patient age and wishes, co-morbidities, salvage status, prior therapies, duration of prior response, exposure to allogeneic SCT, leukemia characteristics, and availability of investigational therapies. Guidelines of salvage therapies offered at our institution are detailed below.

In young/fit patients with AML and failure or progression on 3 + 7 regimens, therapies that include high-dose cytarabine provide good results. Using the FLAG-IDA plus venetoclax regimen in 25 patients in Salvage 1, the marrow CR rate was 65% and 1-year survival 52%^115^. The combination of HMA therapy (azacitidine, decitabine) plus venetoclax may help patients not previously exposed to either agent. For patients post frontline high-dose cytarabine-based regimens (FAI-FLAG/IDA, CLIA) who are in first relapse with a first CR duration of 12 months or longer, we still offer the high-dose cytarabine-based regimens (FLAG-IDA, CIA, CLIA, twice daily fludarabine + cytarabine)^170^ in combination with novel targeted therapies as indicated (venetoclax, FLT3 or IDH inhibitors). In salvage situations, repeating the molecular studies for *FLT3, IDH 1–2*, and *TP53* mutations may identify the emergence of resistant clones with these mutations. Patients may then become candidates for targeted inhibitors-based therapies. Patients in second salvage or beyond are offered phase 1–2 investigational approaches.

Patients achieving subsequent CR should be considered for allogeneic SCT immediately, provided they understand the procedure risks, expected mortality rates, and expected (low) rate of long-term survival. Along these lines, we are investigating a regimen of sequential intensive chemotherapy, with the application of allogeneic SCT at the time of marrow aplasia (Day 21–35 of chemotherapy) rather than after achievement of CR (which is of low probability; <10–20% in most such situations).

### FLT3 inhibitors in AML salvage

Gilteritinib (SP 2215) is a potent type-1 FLT3 inhibitor (dual FLT3-AXL inhibitor) with excellent selectivity against *FLT3* mutations (both *FLT3-*ITD and FLT3-TKD mutations). Gilteritinib 120 mg daily produced CRc (composite CR) rates of 45–50% as a single agent in relapsed/refractory *FLT3-*mutated AML patients^171^. The phase 3 pivotal ADMIRAL trial randomized (2:1) 371 patients with relapsed *FLT3*-mutated AML to gilteritinib 120 mg daily (*n* = 247) or investigator choice salvage chemotherapy (both high- and low-dose chemotherapy) (*n* = 124)^164^. Gilteritinib therapy resulted in a significantly longer survival (median survival 9.3 versus 5.6 months; hazard ratio 0.637; *p* = 0.0007). It was also associated with a higher rates of CR (21% versus 11%; *p* = 0.013), CR/CRh rate (34% versus 15%), and CRc rate (54% vesus 22%)^164^. This led to the FDA approval of single-agent gilteritinib as salvage therapy of *FLT3*-mutated AML. Ongoing studies are combining gilteritinib with HMA therapy and with intensive chemotherapy, as well as with venetoclax in frontline, salvage, and maintenance strategies in AML.

Combination therapy with agents that induce apoptosis may enhance cytotoxicity against *FLT3-*mutated and wild-type clones and potentially delay or prevent drug resistance to FLT3 inhibitor-based therapies. Preclinical data indicated strong synergism between venetoclax and FLT3 inhibitors. An ongoing phase IB study is evaluating the combination of venetoclax and gilteritinib (NCT03625505) in refractory-relapsed AML (most patients with prior exposure to FLT3 inhibitors). Currently, 31 of 37 patients (84%) treated achieved marrow CR; the median duration of response has not been reached^172^. A triplet-therapy combining azacitidine,venetoclax and gilteritinib in older AML is ongoing.

### Isocitrate dehydrogenase inhibitors in AML salvage

The *IDH 1–2* mutations induce neomorphic IDH enzyme activity, which results in aberrant production of the onco-metabolite 2-hydroxyglutarate (2-HG). The 2-HG competitively inhibits alpha-ketoglutarate (αKG), and leads to dyregulated epigenetic function, a hypermethylated phenotype, and a block in maturation, leading to AML tumorigenesis^173^.

Enasidenib, formerly AG221, is an orally bioavailable small molecule inhibitor of mutant *IDH2*, which is FDA approved for the treatment of relapsed-refractory *IDH2*-mutated AML at a dose of 100 mg orally continuously daily. The FDA approval was based on the results of the Phase1–2 trial in 176 patients with relapsed-refractory *IDH2*-mutated AML. Enasidenib therapy resulted in an overall response rate of 41%, a CR/CRh rate of 23%, a median response duration of 5.8 months, and a median survival of 9.3 months. When used as monotherapy, patients with RAS pathway co-mutations and/or high mutational burden (>6 mutations) were less likely to respond^173,174^, suggesting the importance of combination therapy (under evaluation in both newly diagnosed and relapsed *IDH2*-mutated AML). In a randomized Phase 2 study in newly diagnosed *IDH2*-mutated AML of azacitidine + enasidenib compared with enasidenib alone, the combination resulted in a significantly higher CR rate (53% versus 12%) and overall response rate (71% versus 42%), and a trend for improved EFS (17 months versus 11 months). The overall median survival was impressive, 22 months, but similar in both arms, likely because of the availability of effective salvage^175^.

Ivosidenib, formerly AG120, is a selective small molecule inhibitor of *IDH1*. Ivosidenib 500 mg daily was approved by the FDA for the treatment of relapsed-refractory *IDH1*-mutated AML (as well frontline therapy of *IDH1*-mutated AML in patients unfit for intensive chemotherapy) based on the results of the Phase 1–2 clinical trial evaluating 179 patients. In this study, ivosidenib produced an overall response rate of 42%, a CR/CRh of 30%, a CR of 22%, and a median survival of 8.8 months^176^. Similar to enasidenib, mutations in the RTK pathway (i.e., RAS, PTPN11 and FLT3 mutations) were associated with a lower response rate to ivosidenib monotherapy^177^. A trial of ivosidenib + venetoclax + azacitidine is currently ongoing in newly diagnosed and relapsed *IDH1-*mutated AML.

## Expanding on topics of interest in AML

### Polo-like-1 kinase inhibitors

Polo-like kinase-1 (PLK-1) belongs to a family of serine-threonine kinases and plays an important role in centrosome maturation, spindle formation, and cytokinesis during mitosis. It is highly expressed in leukemic cells. Volasertib, a small molecule serine-threonine inhibitor, binds competitively to the kinase ATP-binding pocket and inhibits its enzymatic activity at low nanomolar concentrations. It also inhibits two related PLKs, PLK-2, and PLK-3. The encouraging data from preclinical and phase 1–2 trials led to a phase 2 randomized study of low-dose cytarabine with and without volasertib in patients with AML not suitable for frontline intensive chemotherapy. Among 87 patients randomized (median age 75 years), the addition of volasertib led to a higher overall response rate (31% versus 13.3%; *p* = 0.052) and a longer median survival (8.0 versus 5.2 months; hazard ratio 0.63; *p* = 0.047)^178^. Unfortunately, the phase 3 pivotal trial comparing low-dose cytarabine with or without volasertib in older patients with newly diagnosed AML not eligible for intensive chemotherapy (NCT 01721876) did not meet the study endpoints. The status of volasertib is uncertain, but other presumably better PLK1 inhibitors (such as onvansertib)^179^ are under development.

### Antibodies targeting AML surface molecules

Monoclonal antibodies targeting cluster designation (CD) surface molecules CD33, CD123, CD70, CLL1 (or CLEC12a), TIM3, WT1 and others, may result in important anti-AML efficacy. These antibodies may be unconjugated, conjugated to immunotoxins, or bispecific antibodies (BiTEs) directing killer CD3 T-cells (linking to T-cell CD3) to the AML CD surface molecules.

Unconjugated monoclonal antibodies have so far had little success in AML, as shown with CD33 unconjugated antibodies. A pilot study of azacitidine plus cusatuzumab (monoclonal unconjugated antibody-targeting CD70) was promising^180^. Studies of cusatuzumab combination with azacitidine and/or venetoclax are ongoing.

Monoclonal antibodies conjugated to immunotoxins have had some success, as shown by the experience with GO. Some studies with CD33 and CD123 monoclonal antibodies (e.g., SGN-33A [vadastuxumab], a humanized anti-CD33 monoclonal antibody conjugated to pyrrolo-benzodiazepine) have shown excessive myelosuppression and mortality, resulting in abandoning the drug development. IMGN632 is a CD123 antibody conjugated to an alkyl-benzodiazepine. As a single-agent, IMGN632 was evaluated in 74 patients (67 AML, 7 blastic plasma-dendritic cell neoplasm [BPDCN]). Among 66 evaluable patients with AML, 55% had a reduction in bone marrow blasts, and 20% achieved a CR/CRi across a range of IMGN632 doses (0.045 to 0.3 mg/kg per course). Among seven patients with BPDCN, three (43%) achieved a CR/CRi. IMGN632 monotherapy is being evaluated in patients with relapsed-refractory BPDCN and MRD-positive AML. Combinations of IMGN632 with azacitidine and/or venetoclax are under evaluation in AML (NCT04086264)^181^.

Ongoing studies are evaluating the delivery of radioisotopes using AML surface antigen-targeting antibodies. The clinically most advanced among these is the use of CD45-targeted antibodies (e.g., Iomab-B or 90Y-BC8-DOTA)^182,183^. As CD45 is ubiquitously expressed in the hematopoietic system, CD45-targeting may lead to significant myeloablation and such approaches are studied as part of pre-SCT conditioning in transplant-eligible patients. A randomized phase III study evaluating this approach with Iomab-B versus investigator choice salvage therapy prior to SCT in patients with relapsed-refractory AML is ongoing (NCT02665065).

The bispecific T-cell engaging antibody (BITE) technology utilizes bispecific antibody constructs that recruit CD3-effector T cells to target tumor cells (CD33, CD123, and also CD70 in the case of AML). Several AML-targeted BiTEs are under development in AML, including flotetuzumab, AMG-330, AMG673, AMG 427, XmAb14045, AMV564. Several have shown modest activity (response rates 20 to 30%) and were associated with the predicted toxicities (fever, hypotension, cytokine release syndrome). A potential area of research interest is exploring their efficacy in the setting of AML in CR with MRD-positive disease (as was done with blinatumomab in ALL).

### CAR-T cellular therapy in AML

The success of immunotherapy in cancer led to renewed interest in developing immune-based strategies in AML, including antibody-based (discussed earlier) and cellular therapy. Trials of chimeric antigen receptor (CAR)-T cells are ongoing including autologous and allogeneic CART cells (targeting CD123, CD33, and CLL1) followed by allogeneic SCT.

### Summary

Many of the hopeful predictions outlined in the AML summary of 2016 are now therapeutic realities: GO, venetoclax, FLT3 inhibitors (midostaurin, gilteritib), IDH inhibitors (ivosidenib, enasidenib), CPX-351, glasdegib, oral decitabine, and oral azacitidine. Others may soon be (quizartinib, APR246, magrolimab, menin inhibitors). The wealth of positive data allows reconsideration of what might soon be new standards of care during induction-consolidation-SCT-maintenance in younger and older patients with AML.
